# Testing the Usability of Digital Educational Games for Encouraging Smoking Cessation

**DOI:** 10.3390/ijerph17082695

**Published:** 2020-04-14

**Authors:** Jong-Long Guo, Hsiao-Pei Hsu, Mei-Hsun Lin, Cheng-Yu Lin, Chiu-Mieh Huang

**Affiliations:** 1 Department of Health Promotion and Health Education, College of Education, National Taiwan Normal University, Taipei 106, Taiwan; jonglong@ntnu.edu.tw; 2 Institute of Clinical Nursing, School of Nursing, National Yang-Ming University, Taipei 112, Taiwan; sandylucy616@ym.edu.tw; 3 Cathay Healthcare Management, Taipei 106, Taiwan; maniahsun@cathay-hcm.com.tw; 4 Department of Radio, Television & Film, Shih Hsin University, Taipei 116, Taiwan; cyou.lin@msa.hinet.net

**Keywords:** usability test, digital educational games, smoking cessation

## Abstract

This study, using an observational design, assessed the effect of digital educational games on students’ intention to quit smoking. Specifically, a series of digital games were developed to encourage smoking cessation and enhance students’ engagement with the material. Three determinants of engagement were tested: perceived ease of use, perceived usefulness, and perceived satisfaction. Usability assessments were performed using a structured questionnaire and usability-testing software (Morae). Most students reported that the games were easy to use (73.3–93.3%), useful (60.0–83.3%) and satisfactory (66.7–70.0%). After using the games, approximately half (46.7–53.3%) reported that they intended to quit smoking. After controlling for gender and age, multiple regression analysis revealed that only perceived usefulness statistically significantly contributed to intention to quit (β = 2.38, *p* < 0.05). ‘Taiko Drumming’ showed the highest number of mouse clicks (67.23), and the lowest time between inputs (7.88 s). It also received the most positive feedback (17 marks). These findings show that integrating learners’ experiences into the development of learning material can improve learning effectiveness.

## 1. Introduction

Tobacco use is the leading cause of preventable death worldwide, and is associated with malignant tumours, cardiovascular diseases, and chronic diseases [1,2]. Symptoms of dependence commonly appear soon after commencement of intermittent smoking [3], and 25% of youths experience symptoms of dependence within 23 months of tobacco-use onset [3]. Nicotine dependence in adolescence is an important public-health issue, and as such early smoking and dependence may predict smoking-related diseases in later adulthood [4]. Individuals who develop dependency in adolescence show more negative health symptoms in early adulthood when compared to non-dependent smokers and non-smokers [5]. Approximately one-third of novice smokers escalated to higher levels of consumption relatively quickly after initiating tobacco use [6]. A longitudinal study of smoking trajectories suggested that novice smokers should be targeted for early tobacco control intervention to prevent development of nicotine dependence and sustained smoking [7].

In order to achieve the goal of tobacco control, Taiwan implemented the “Tobacco Hazards Prevention Act” in 2009. In the past decade, the smoking rate of senior high school students has decreased from 14.8% in 2009 to 8.0% in 2018 [8]. However, the smoking rate (2.8%) of junior high school students was less than that of senior high school students [8]. This finding implied that the number of students smoking sharply increased from junior high school to senior high school. Thus, it was important to provide educational interventions to help young students quit smoking. Cessation programmes on campus would be beneficial for preventing students from continuing smoking into adulthood.

According to the American Lung Association, quitting smoking often requires multiple attempts; further, 70–80% of smokers desire to quit, and one-third have made at least three serious attempts to do so [9]. This suggests that quitting smoking is extremely difficult. Possible reasons include smokers’ physical addiction to nicotine, and that smokers link smoking with many social activities [10,11]. In an attempt to prevent smoking students from developing nicotine dependence and, consequently, adverse health effects, the Taiwan Tobacco Hazards Prevention Act stipulates that smokers under 18 years of age must receive a three-hour educational course on quitting smoking [10]. Schools are required to provide these educational courses; however, school personnel may experience difficulties sufficiently engaging and educating students within the short period provided (three hours).

To motivate students to quit smoking, it is important to present engaging content as soon as possible; a lack of such engagement may undermine the intervention’s effectiveness [11]. Enhancing engagement also increases the likelihood that the users will develop motivation to perform the target behaviour (in this case, smoking cessation). Thus, enhancing student engagement may have a favourable impact on learning outcomes [12]. Educational games have been found to quickly engage young students [13], meaning digital materials that feature games may be an effective educational tool for helping school staff conduct smoking-cessation courses. Notably, to achieve user engagement, usability testing is strongly recommended [14]. Usability testing also provides opportunities to observe students as they interact with the digital material, allowing developers to identify and eliminate potential problems.

In accordance with the above reasoning, we hypothesized that higher user engagement results in stronger motivation to quit. Three determinants of engagement were considered: perceived ease of use (the students’ perceptions regarding whether the digital material was easy to use [15]), perceived usefulness (whether the digital material enhanced learning efficiency [12,16]), and perceived satisfaction (whether the students’ expectations were confirmed by their use experiences [15]).

The purpose of this study was: (1) to assess the usability of digital educational games concerning smoking cessation, and (2) to assess participants’ intention to quit smoking after playing the digital educational games.

## 2. Method

### 2.1. Participants and Recruitment

The study featured an observational design. The participants were regular smokers who were (a) currently vocational high-school students, (b) required to attend a smoking-cessation course, (c) not pregnant, (d) not diagnosed with any major chronic diseases (e.g., asthma, heart disease, or diabetes), (d) willing to comply with verbal instructions, and (e) able to provide written informed consent.

All participants were recruited from three vocational high schools in northern Taiwan. For each school, the research team met with personnel who were responsible for providing the smoking-cessation courses. During these meetings, the digital educational games were distributed and potential barriers regarding their implementation were discussed. The school personnel then explained the research project to the students and distributed an information sheet. Student smokers who were willing to participate were informed that their on-screen activities would be monitored and recorded while they played the games.

### 2.2. Design of the Digital Educational Games

The digital educational games were designed to be motivational tools for encouraging smoking cessation and, through their game format, to be engaging for students. The materials represented six topics: (a) increasing awareness of high-risk situations, (b) addressing withdrawal syndrome, (c) negotiating cessation barriers, (d) encouraging oneself through reinforcing one’s resolve to make the right decision, (e) addressing negative responses associated with hunger, anger, loneliness, and fatigue, and (f) evaluating the strength of various stressors. These course topics were derived from previous smoking-cessation programs [15,17,18]. To advance students’ engagement, an educational game was designed for each topic (Table 1). Specifically, the tasks in these digital games involved sticky notes (this game is referred to as ‘Sticky Notes’ hereinafter), puzzle pieces (‘Puzzle Pieces’), throwing cessation barriers into a virtual trash can (‘Trash Can’), Taiko drumming (‘Taiko Drumming’), scratch cards (‘Scratch Cards’) and click-and-drag (‘Click-and-Drag’) actions, respectively.

### 2.3. Research Instruments

We collected participants’ background information, including gender, age, school level at which they had commenced smoking, whether they had ever attempted to quit, and the smoking status of their parents and friends, respectively. The following structured questionnaires were then administered.

### 2.4. Measures

#### 2.4.1. Nicotine Dependence

The Fagerström Test for Nicotine Dependence (FTND) has been found to be relatively suitable for screening for smoking behaviour in habitual adolescent smokers [6]. This scale contains six items, with total scores ranging from 0 to 10 points. Scores of four and above represent moderate to severe dependence (higher scores indicate stronger dependence [19]). The FTND has previously been used in a study of youth smokers in Taiwan, and exhibited satisfactory validity and reliability [6,20]. The scale evaluates the quantity of cigarette consumption, compulsion to use, and dependence. Example items are: ‘How many cigarettes per day do you smoke?’ and ‘How soon after you wake up do you smoke the first cigarette?’

#### 2.4.2. Usability Assessment: Determinants of Engagement

Perceived ease of use. Perceived ease of use was defined as the extent to which the students perceived the digital material as being easy to operate. This was measured using four items (e.g., ‘The interfaces for the digital educational games are easy to understand’), which were scored using a five-point Likert scale ranging from 1 (‘strongly disagree’) to 5 (‘strongly agree’). For this study, the Cronbach’s α coefficient was 0.79.

Perceived usefulness. Perceived usefulness was defined as the extent to which the students perceived the digital material as being helpful for smoking cessation. This was measured using four items (e.g., ‘I felt that the digital educational games were helpful for increasing my awareness of cessation barriers’), which were scored using a five-point Likert scale ranging from 1 (‘strongly disagree’) to 5 (‘strongly agree’). For this study, the Cronbach’s α coefficient was 0.81.

Perceived satisfaction. Perceived satisfaction was defined as the students’ satisfaction with the design composition and layout, the content, the game, and the operation duration. It was measured using a five-point Likert scale ranging from 1 (‘strongly disagree’) to 5 (‘strongly agree’). For this study, the Cronbach’s α coefficient was 0.83.

Intention to quit. Intention to quit was defined as whether the students intended to quit smoking by using the knowledge they had gained from digital material as a reference. This was measured using four items (e.g., ‘I would consider quitting smoking by using the content of the digital educational games as a reference’), which were scored using a five-point Likert scale ranging from 1 (‘strongly disagree’) to 5 (‘strongly agree’). For this study, the Cronbach’s α coefficient was 0.90.

#### 2.4.3. Usability Assessment: Morae

Morae is a software that helps designers gain insights into users’ experiences. Morae comprises three components: ‘recorder’, ‘observer’, and ‘manager’. The recorder application allowed us to monitor users’ on-screen actions, including keyboard and mouse activity. The observer application allowed us to create markers to note and code the users’ responses; these markers then appear in the recorder file. We then imported the file into the manager application, which allowed us to analyze the collected data and create graphs.

When using observer to mark students’ reactions toward the digital materials, reactions were grouped into six types: (1) operation mistakes (when students pressed the ‘next’ button, but had not yet completed the task requirement; when students misunderstood the task requirement and pressed an incorrect button); (2) hesitations/pauses (when students exhibited an obvious hesitation/pause without performing any on-screen activities); (3) asking questions (when students asked questions aloud; some students would ask questions when they made operation mistakes or hesitated/paused); (4) positive feedback (when students expressed their enjoyment of the games, or laughed aloud); (5) negative feedback (when students verbally stated that the games were boring, not enjoyable, childish, or involved too many tasks); (6) System error (when the software did not function correctly).

### 2.5. Data-Collection Procedure

Once students provided approval and written consent, they were directed to sit at a laptop and access the digital material, which was presented in an environment monitored by the Morae software. Students were informed that their on-screen activities and reactions to the material would be recorded, and they provided consent. Research staff were present while the students completed the six sections, but did not provide any oral instructions. Students could ask questions and freely express their opinions at any time. Once students completed the digital material, they were provided with a structured questionnaire concerning the games’ usability.

### 2.6. Data Analysis

Statistical analysis was performed using SPSS 20.0 (SPSS, Inc., Chicago, IL, USA). The participants’ characteristics were described using percentages. Means (standard deviations) and percentages were used to represent the determinant variables. Pearson’s correlation analyses and multiple regression analyses were used to examine the associations between determinants of engagement and intention to quit. In addition, the on-screen activities were reported using Morae manager. Two methods were used for diagnosing multicollinearity—variance inflation factor (VIF) and tolerance. VIFs should not exceed 6 [21]. When the value of tolerance is less than 0.1 [22], multicollinearity can become a problem. In this study, the VIFs were less than 6 (from 2.40 to 2.49) and the tolerance values were higher than 0.1 (from 0.40 to 0.42) for the study variables. Therefore, multicollinearity did not appear to be a problem in the regression analysis. In addition, the on-screen activities were reported using Morae manager.

### 2.7. Ethical Considerations

The study protocol was approved by the institutional review boards of the National Taiwan Normal University. All participants were volunteers and had signed an informed consent form. The participants were permitted to withdraw from the study at any point.

## 3. Result

As Table 2 indicates, the participants comprised 22 males and eight females; their mean age was 18.57 years. Over half had initiated smoking while they were in junior high school (56.7%), and over half had attempted to quit (53.3%). Most had family members who were smokers (76%), and all had friends who were smokers (100%). Over half (70.0%) reported a moderate to severe level of nicotine dependence, as identified by an FTND score of ≥4.

Table 3 shows the distribution of the determinants of engagement and intention to quit. Most students agreed or strongly agreed that the digital educational games were easy to use (73.3–93.3%), useful (60.0–83.3%), and satisfactory (66.7–70.0%). Approximately half (46.7–53.3%) agreed or strongly agreed that they had developed an intention to quit after using the games.

Pearson’s correlation analysis revealed that the more the students perceived the games as easy to use (r = 0.40, *p* < 0.05), useful (r = 0.56, *p* < 0.01), and satisfactory (r = 0.40, *p* < 0.05), the higher their level of intention to quit. However, after controlling for gender and age, multiple regression analysis revealed that only perceived usefulness statistically significantly contributed to intention to quit (β = 2.38, *p* < 0.05).

**Figure 1:**
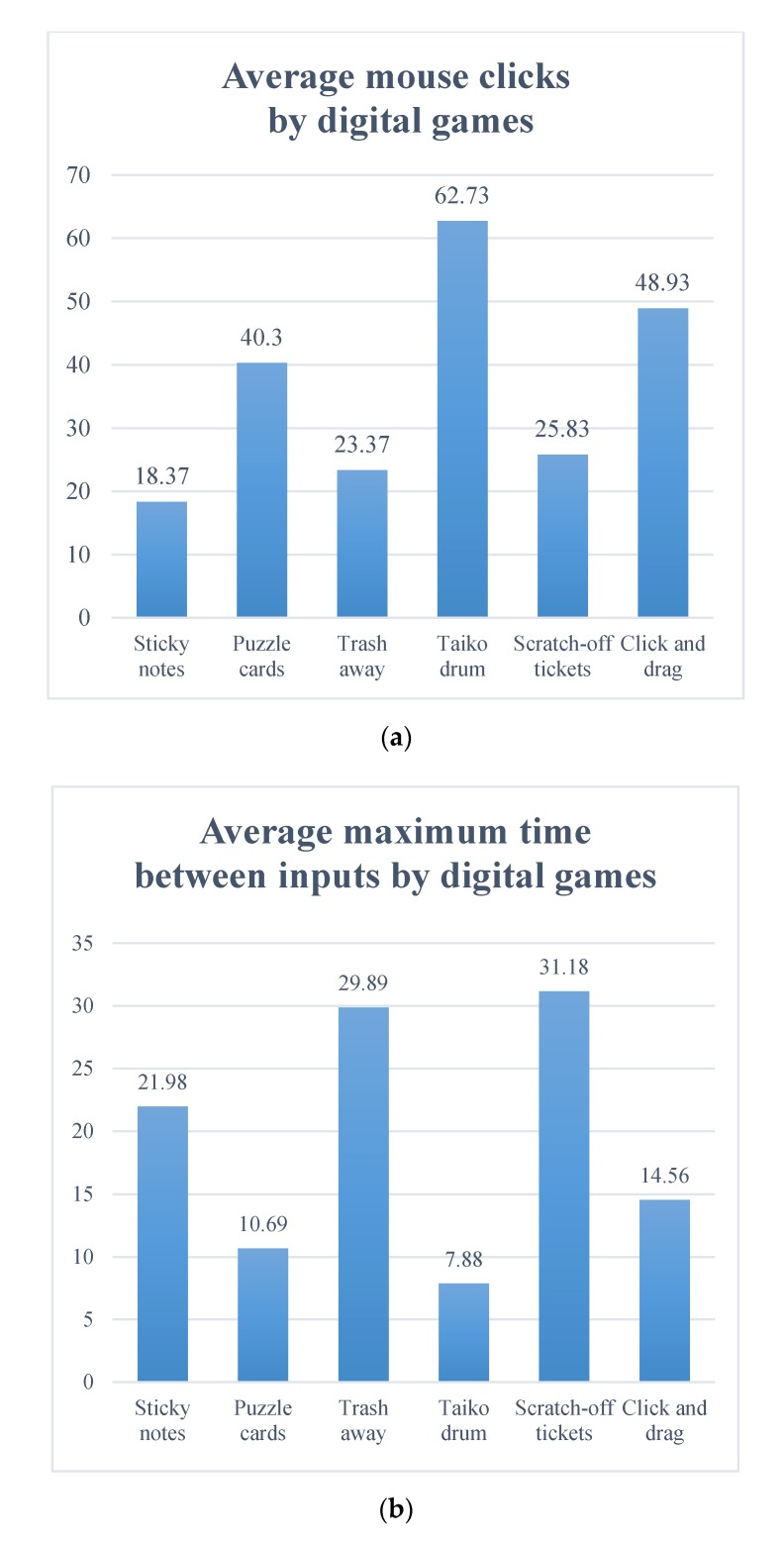
On-screen activities for the six digital games. (**a**): mouse clicks (count); (**b**) maximum time between inputs (seconds).

In addition to self-reported data, several graphs were generated through Morae. Observation of the students’ on-screen activities revealed an opposite tendency between ‘mouse clicks’ and ‘time between inputs’. Two patterns of on-screen activities were found across the six games. The first pattern was, as expected: the higher the number of mouse clicks, the less time between inputs. This pattern was observed for ‘Puzzle Pieces’, ‘Taiko Drumming’, and ‘Click-and-Drag’. The second pattern was as follows: the lower the number of mouse clicks, the more time between inputs. This pattern was observed for ‘Sticky Note’, ‘Trash Can’, and ‘Scratch Card’ tasks. Overall, ‘Taiko Drumming’ showed the highest number of mouse clicks (67.23), and the lowest time between inputs (7.88 s). Interestingly, the sequential arrangement of the two patterns showed alternating changes across the games (Figure 1); we did not consider this when originally arranging the games.

After recording, we used observers to note the students’ reactions toward the digital games (Table 4). ‘Asking questions’ was the most-noted reaction (63 marks), while ‘operation mistakes’ was the least-noted reaction (two). The games during which the participants asked the most questions were ‘Sticky Notes’ (12 marks), ‘Trash Can’ (21), and ‘Scratch Cards’ (15). Similarly, these games were associated with more hesitations/pauses (12, 11, and 21 marks for ‘Sticky Notes’, ‘Trash Can’, and ‘Scratch Cards’, respectively). ‘Taiko Drumming’ received the most positive feedback (17 marks); however, in contrast, some students thought that ‘Taiko Drumming’ was too childish, and gave negative feedback (three marks). Few system errors were recorded (two marks).

## 4. Discussion

In this study, user experience was defined in terms of engagement and motivation. To obtain effective e-learning, the manner by which the content is delivered to the learners is important [19,23]. The six educational games examined in this study were designed to enhance the students’ knowledge-building through engaging them with the material. Our results revealed that most of the students perceived the digital materials as easy to use, useful, and satisfactory. Presenting the same messages in plain text would not have induced the same level of interest [24,25]. Providing such education through simple interactive games is beneficial for learners’ focus and concentration [26].

We found, among a group largely comprising individuals with moderate to heavy nicotine dependence (70%), that the digital educational games enhanced motivation to quit smoking. Approximately half of the participants agreed or strongly agreed that they intended to quit smoking by using the material provided in the games as a reference. Intention to quit is essential for initiating behavioural changes [27]. Behavioural intention, which was theoretically used as a proxy for behaviour, was reported to have a causal impact on behaviour [28]. Behavioural intention does not necessarily lead to actual behavioural practice. Scholars have suggested that there is a gap between intention and behaviour [29]. However, some behavioural science studies have provided evidence of a positive association between intention and behaviour. A meta-analysis of 47 experimental studies examined whether changes in behavioural intention engendered behavioural change [30]. The findings showed that a medium-to-large change in behavioural intention (d = 0.66) resulted in a small-to-medium change in behaviour (d = 0.36) [30]. Additionally, Sheeran (2002) presented a meta-analysis of the intention-behaviour relationship. The correlation between intention and behaviour derived from these studies was 0.53 [31].

In the context of quitting smoking, a study revealed that a positive association was found between intention and behaviour (*p* = 0.018) [32]. Our findings indicate that, for cases in which human resources and time are limited, the provision of digital-based interventions may increase the accessibility of mandatory smoking-cessation services as well as the intention to use the knowledge provided. However, caution is necessary while generalising the findings. Although student participants expressed their intentions to quit smoking, the school staff should be aware of the discrepancy between intentions and actions. Providing smoking cessation education alone is not sufficient for students to act on quitting smoking.

For the games, participants of all genders, ages, and levels of nicotine dependence showed acceptance of the games. The participants scored high on these study variables, which indicates a certain degree of general acceptance about the educational games. The participants’ background characteristics were not associated with perceived ease of use, usefulness, or satisfaction. Due to a ceiling effect, the results can be interpreted as not having sufficient power to detect group differences.

Our results highlight a potential new field for the application of digital education games. We also found that the determinants of engagement (the perceived ease of use, usefulness, and satisfaction) were statistically significantly associated with intention to quit smoking. These results are similar to previous findings regarding the influence of usability, usefulness, and satisfaction on behavioural intentions [33,34]. The Keller’s ARCS model is an instructional design that focuses on the motivational aspects of learning [35]. According to ARCS Model, four essential components of attention (A), relevance (R), and confidence (C), and satisfaction (S) can be used for designing motivating instruction during learning process [35,36]. However, in this study satisfaction had a lower influence upon intention to quit when compared to perceived usefulness (which, among the three determinants of engagement, was the most evident contributor to intention to quit). Participants may have felt that the digital content could be helpful for future smoking cessation, and been motivated by a sense of relevance [14]. The more students perceived the content as helpful for learning about smoking-cessation barriers, processes, and strategies, the more they considered quitting by using the educational content as a reference.

In this study, most students agreed/strongly agreed that the games were easy to use. A previous study suggested that a good interface design increases convenience by making computers more user-friendly [37]. However, our participants’ actual experience with the games may not have been as good as we hypothesized. We received similar amounts of positive and negative feedback from the students. Although students felt that the games were easy to use, this did not guarantee error-free operation.

To assess usability, we integrated two instruments, a structured questionnaire and Morae. This combined approach deepened our understanding of the users’ experiences. Our results highlight that a strong understanding of users’ experiences is important for digital-media design. According to Morae observer, the games with the highest numbers of markers/notes were associated with more questions and hesitations/pauses among the students. Specifically, these games were ‘Sticky Notes’, ‘Trash Can’, and ‘Scratch Cards’. As expected, these three games also showed more time between inputs. In technology-enhanced learning, different types of digital games may produce different impacts [37]. We also observed a pattern comprising an association between negative feedback and asking questions and hesitating/pausing. A possible explanation for this is that an absence of interruptions during tasks is essential for a satisfying experience. However, asking questions and hesitating/pausing could also be a manifestation of focusing attention. In other words, this could indicate that students were endeavoring to resolve the problems they encountered. To advance the game design for education, it was necessary to identify game elements that influenced students’ learning [38]. This was considered beneficial for the game design comprising a collection of different scene activities in which the player works on the educational task or resolving challenges [39]. This study applied Morae software for documentation during game implementation. The documentation described different aspects of the participants while playing the same game. However, the qualitative aspects of participants’ experiences were not captured through Morae documentation. Therefore, it was important to incorporate qualitative interviews as part of data collection for validating the previous hypothetical reasoning.

Although two patterns of screening activities were observed across the six digital games, the positive and negative feedback did not seem to be associated with these patterns. The findings in this regard suggested that the participants evaluated the games based on a more complicated framework than input patterns alone. The most popular educational game was ‘Taiko Drumming’. Studies have highlighted three essential aspects for a good gaming environment: clear and concise instructions, challenging tasks, and player control over several gaming options [36,37]. ‘Taiko Drumming’ featured familiar scenarios for most of the participants, and the challenges involved mouse-clicking on correct musical notes. These traits may have increased the students’ interest and engagement. Moreover, some participants reported that the music enhanced their focus on the challenge, and that the cheers they received for correct answers helped them to feel a sense of achievement.

### Limitations

Despite the interesting insights obtained through this research, there are some limitations. The first is that this study applied a nonprobability sampling strategy, which may have limited the inferences that can be drawn for a larger population. The exploration of students’ experiences can be used for the improvement of digital education games, rather than for generalizing the statistical findings to a population. Second, the small sample size may have resulted in insufficient power to detect the associations among variables. It would be beneficial to include more samples in future studies. Third, students were aware that their on-screen activities would be monitored and recorded, which could have influenced their responses. However, it is reasonable to assume that most students felt sufficiently secure to express their perspectives, because the highest reaction marks concerned asking questions, and the marks for positive and negative feedback were almost equal.

## 5. Conclusions

Our findings suggest that digital educational games represent an alternative strategy for engaging young students. When time and human-resource limitations are present, school staff could adopt such games to enhance smoking students’ interest in smoking-cessation-related materials, as well as their intention to quit. In particular, digital educational games are a possible means of motivating students with moderate to severe levels of nicotine dependence (as identified using FTND) to quit smoking.

When designing digital educational games for encouraging smoking cessation, focusing solely on ease of use may not be sufficient. Perceived usefulness should be prioritized, as this perception is the most evident influencing factor. Material that is relevant to students’ experiences should also be incorporated into the game-design process. In our study, the students evaluated the digital games based on more than just input patterns; features such as familiar scenarios, challenging tasks, and cheers for achievements were also important.

During the development of digital educational games, the exploration of students’ experience would be useful for improvement. Our findings provided information concerning the participants’ interaction with the digital games, as well as their perceptions of the games. Overall, such information can help developers gain insight into users’ experiences.

## Figures and Tables

**Table 1:** Content of the digital material for encouraging smoking cessation.

Topic	Operation Instructions	Example of the Digital Game
(a) Increasing awareness of high-risk situations	Students were provided with a group of sticky notes that featured descriptions of situations in which they may be tempted to smoke. The students arranged the order of the sticky notes based on the severity of the temptation associated with each situation.	Sticky Notes 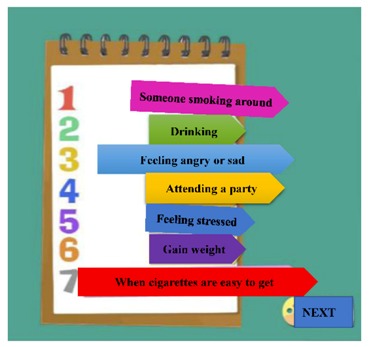
(b) Coping with withdrawal syndrome	Students were provided with some puzzle pieces on which coping strategies for withdrawal syndrome were described. They arranged these puzzle pieces to create a unified shape.	Puzzle Pieces 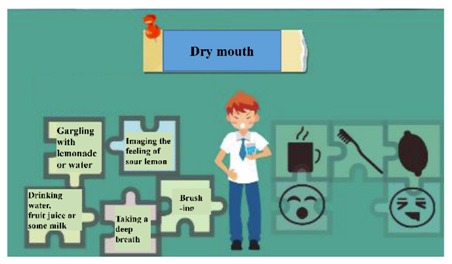
(c) Negotiating cessation barriers	Students were encouraged to overcome cessation barriers. Using trash bags as a metaphor, students threw the bags into the garbage can to symbolize a determined action. After completing this, possible solutions were presented.	Trash Can 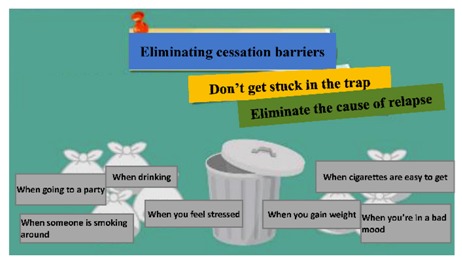
(d) Encouraging oneself through reinforcing one’s resolve to make the right decision	Students drummed in time with the rhythm of a musical piece. As the music continued, sentences encouraging the participant to quit smoking appeared. On-screen cheerleaders celebrated when the students made the right decisions.	Taiko Drumming 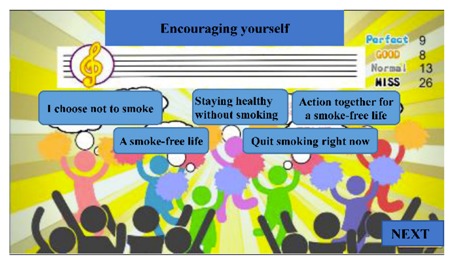
(e) Addressing negative responses associated with hunger, anger, loneliness, and fatigue.	When beginning to try to quit smoking, certain negative stimuli may trigger a relapse, such as hunger, anger, loneliness, and fatigue. This game presented students with scratch cards that featured these triggers. By scratching the cards to reveal the triggers, students’ recognition of these stimuli was enhanced. After completing a scratch card, possible solutions were presented.	Scratch Cards 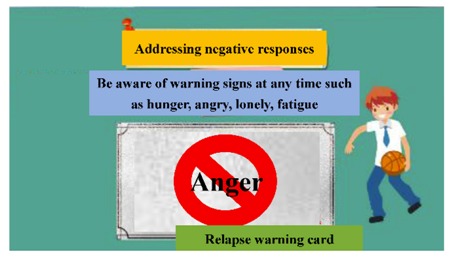
(f) Evaluating the strength of various stressors	Students read a scenario describing a day in the life of a student. After completing the reading, they were presented with a number of stressors corresponding to parts of the scenario. For each stressor, students clicked and dragged points on a scale ranging from 1 to 5; higher numbers indicated a higher level of stress.	Click and Drag 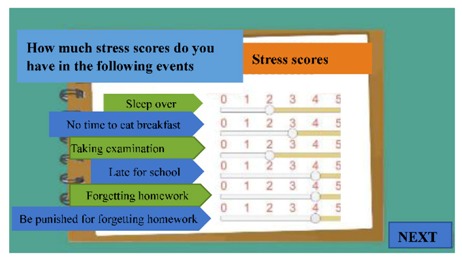

**Table 2:** Students’ background information.

Variable	*n* (%)
Gender	
Male	22 (73.3%)
Female	8 (26.7%)
Age	18.57 ± 2.08
≤17	11 (36.7%)
18	10 (33.3%)
≤19	9 (30.0%)
School level when commencing smoking	
Elementary school	5 (16.7%)
Junior high school	17 (56.7%)
Senior high school	8 (26.7%)
Number of cigarettes smoked per day	
≤10	12 (40.0%)
11–20	9 (30.0%)
≥21	9 (30.0%)
Whether family members smoke	
No	7 (23.3%)
Yes	23 (76.7%)
Whether friends smoke	
No	0 (0.0%)
Yes	30 (100.0%)
Degree of nicotine dependence (FTND score)	
Light (1–3)	9 (30.0%)
Moderate to severe (4–10)	21 (70.0%)

FTND: Fagerström Test for Nicotine Dependence.

**Table 3:** Distribution of the determinants of engagement and students’ intention to quit.

Items	Total (*n* = 30)		Disagree & Strongly Disagree	Neutral	Agree & Strongly Agree
	Mean	SD	%	%	%
Perceived ease of use	17.17	2.63			
The interfaces of the digital educational games are easy to understand.	4.20	0.85	3.3	16.7	80.0
The interfaces of the digital educational games are simple to operate.	4.40	0.86	3.4	13.3	83.3
The content of the digital educational games is clear and comprehensible.	4.47	0.63	0.0	6.7	93.3
I learned how to operate the digital educational games within a short amount of time.	4.10	1.00	3.4	23.3	73.3
Perceived usefulness	15.90	2.83			
I felt that the digital educational games were helpful for increasing my awareness of cessation barriers.	3.83	0.99	3.3	36.7	60.0
I felt that the digital educational games were helpful for understanding the cessation process.	4.10	0.80	0.0	26.7	73.3
I felt that the digital educational games were helpful for learning cessation strategies.	4.20	0.81	3.4	13.3	83.3
In general, I felt that the digital educational games were useful.	3.77	0.94	3.3	36.7	60.0
Perceived satisfaction	15.90	2.83			
I was satisfied with the design, composition, and layout of the digital education games.	3.87	0.97	10.0	23.3	66.7
I was satisfied with the content.	4.00	0.87	3.3	26.7	70.0
I was satisfied with the game.	4.10	0.85	0.0	30.0	70.0
I was satisfied with the time required to operate the games.	3.93	0.79	0.0	33.3	66.7
Intention to quit smoking	14.17	3.43			
I would consider quitting smoking by using the content from the digital educational games as a reference.	3.63	0.96	6.7	40.0	53.3
I would like to attempt to quit this month by using the content of the digital educational games as a reference.	3.53	1.07	13.3	40.0	46.7
I would continue to attempt to quit this year by using the content of the digital educational games as a reference.	3.50	0.86	13.4	33.3	53.3
I would continue to abstain from smoking by using the content of the digital educational games as a reference.	3.50	0.97	13.4	33.3	53.3

**Table 4:** Students’ reactions to the digital educational games.

	Digital Game	Sticky Notes	Puzzle Pieces	Trash Can	Taiko Drumming	Scratch Cards	Click-and-Drag	Total Number of Markers
Identified Markers								
Operation error		1	9	1	5	0	2	18
Hesitations or pauses		12	1	11	2	21	3	50
Asking questions		12	6	21	3	15	6	63
Positive feedback		0	5	1	17	2	1	26
Negative feedback		4	5	3	3	5	7	27
System problems		2	0	0	0	0	0	2
Total number of markers		31	26	37	30	43	19	186

## Abbreviations

FTND: The Fagerström Test for Nicotine Dependence.
